# A 29-year old man with abdominal pain

**DOI:** 10.11604/pamj.2018.29.51.14813

**Published:** 2018-01-19

**Authors:** Petros Ioannou

**Affiliations:** 1 Internal Medicine Department, University Hospital of Heraklion, Crete, Greece

**Keywords:** Splenic infarct, cardiomyopathy, emboli

## Images in medicine

**Figure 1:**
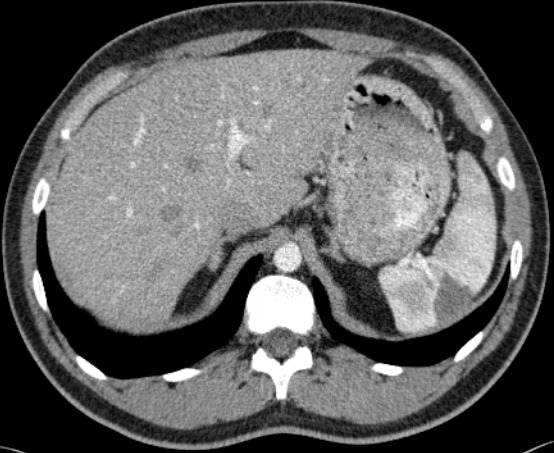
the abdominal computerized tomography with intravenous contrast shows a peripheral hypodense triangular area at the spleen of about 3.2cm x 2.9cm that does not uptake contrast, suggesting a splenic infarct

A 29-year old man with a history of hypertrophic cardiomyopathy with an implanted cardiac defibrillator due to previous ventricular tachycardia and a family history of sudden cardiac death presented to the Emergency Department because of severe left sided abdominal pain that was non-colicky and was not associated with food. The patient denied urinary frequency, hematuria, vomiting or diarrhea and had normal bowel movements. At clinical examination he was afebrile, with a normal blood pressure, a heart rate of about 100bpm while he had left upper quadrant tenderness at the examination of the abdomen, without rebound tenderness, while the abdominal sounds were normal. Complete blood count revealed leukocytosis with a white blood cell count of 13.800 cells, while transaminases, bilirubin, amylase and urine chemistry were normal. An electrocardiogram showed sinus tachycardia. A computed tomography of the thorax and the abdomen with intravenous contrast was performed and revealed an infarct of the spleen as the cause for the left upper quadrant abdominal pain. A representative image is shown in (Figure 1). The patient was admitted and a heart ultrasound revealed the presence of a thrombus in the left ventricle. Splenic infarction is an unusual condition that has been described in almost any age, is mostly associated with hematologic disorders in patients less than 40 years old and with thromboembolic disease in older patients. Other causes are splenic vascular disease, collagen vascular disease, anatomic abnormalities, pancreatic disease and solid tumors.

